# Complete Genome Sequences of Two Akabane Virus Strains Causing Bovine Postnatal Encephalomyelitis in Japan

**DOI:** 10.1128/MRA.00807-20

**Published:** 2020-09-24

**Authors:** Misuzu Okajima, Makoto Ozawa, Isshu Kojima, Hiroaki Shirafuji, Tohru Yanase, Tatsunori Masatani

**Affiliations:** a Transboundary Animal Diseases Research Center, Joint Faculty of Veterinary Medicine, Kagoshima University, Korimoto, Kagoshima, Japan; b Joint Graduate School of Veterinary Medicine, Kagoshima University, Korimoto, Kagoshima, Japan; c Laboratory of Animal Hygiene, Joint Faculty of Veterinary Medicine, Kagoshima University, Korimoto, Kagoshima, Japan; d Kyushu Research Station, National Institute of Animal Health, NARO, Kagoshima, Kagoshima, Japan; KU Leuven

## Abstract

Akabane virus (AKAV) (genus *Orthobunyavirus*, family *Peribunyaviridae*) is an arthropod-borne virus that causes congenital abnormalities in ruminants. Here, we report the complete genome sequences of two AKAV strains causing nonsuppurative encephalomyelitis in cattle by postnatal infection in Japan.

## ANNOUNCEMENT

Akabane virus (AKAV) is an enveloped, tripartite, negative-sense RNA virus of the species Akabane orthobunyavirus (genus *Orthobunyavirus*, family *Peribunyaviridae*). Since it was first isolated from mosquitos in Gunma, Japan, in 1959, it has been widely detected in Australia, Asia, and Africa (1). Currently, >600 sequences of AKAV genomic segments are available in GenBank. AKAV is transmitted by biting midges (*Culicoides* spp.) and causes spontaneous abortions, stillbirths, and congenital malformations in pregnant ruminants (1). Additionally, young or adult cattle infected with some strains of AKAV develop nonsuppurative encephalomyelitis, with neurological symptoms including astasia, ataxia, and opisthotonos (1).

AKAV strains are clustered into four genogroups based on phylogenetic analysis of their medium (M) segments (1, 2). Genogroup I and II strains have been isolated in Japan. Strains of both genogroups cause abortions, stillbirths, and congenital malformations, whereas postnatal encephalomyelitis is caused mostly by genogroup I strains (1). The KM-2/Br/06 strain was isolated in September 2006 in Kumamoto, Japan, from the brain of a cow showing astasia, tachypnea, tremor, and opisthotonos and was classified as genogroup I (2). The FI-1/Br/08 strain was isolated in October 2008 in Fukui, Japan, from the brainstem of a neurologically abnormal cow that had symptoms including astasia, dysstasia, and ataxia (3). The latter strain was the first genogroup II strain implicated in bovine postnatal encephalomyelitis. Here, we report the complete genome sequences of KM-2/Br/06 and FI-1/Br/08, covering their three genomic RNA segments (large [L], M, and small [S] segments).

Both virus strains were propagated in HmLu-1 cells. Their genomic RNA was extracted using the QIAamp virus RNA minikit (Qiagen, Hilden, Germany). Reverse transcription-PCR was performed using SuperScript IV reverse transcriptase (Thermo Fisher Scientific, Waltham, MA, USA) and PrimeSTAR GXL DNA polymerase (TaKaRa Bio, Shiga, Japan). Primer sequences are available on request. The 5ʹ- and 3ʹ-terminal sequences of all of the genomic RNA segments were determined using 5′ and 3′ rapid amplification of cDNA ends (RACE) (4). The sequences were generated in an Applied Biosystems 3730xl DNA analyzer or an Applied Biosystems 3130xl genetic analyzer (Thermo Fisher Scientific) by a DNA sequencing service (FASMAC, Kanagawa, Japan). For both strains, we obtained 14, 9, and 3 Sanger reads for the L, M, and S segments, respectively. Reads were visually assembled using ApE (https://jorgensen.biology.utah.edu/wayned/ape), with the OBE-1 strain (the genogroup II strain that is the progenitor of the vaccine strain used in Japan) as the reference (S segment, GenBank accession number NC_009896; M segment, NC_009895; L segment, NC_009894).

The full-length S, M, and L segments of KM-2/Br/06 are 856, 4,309, and 6,869 nucleotides (nt), respectively, whereas those of FI-1/Br/06 are 867, 4,308, and 6,867 nt, respectively. The GC contents of the segments were also determined (Table 1). The nucleotide identities were compared against each segment of the AKAV OBE-1 strain (Table 1).

**TABLE 1:** Full genome information for each strain and similarity to the AKAV OBE-1 strain

Strain	L segment				M segment				S segment			
	Size (nt)	GC content (%)	Similarity to OBE-1 (%)	GenBank accession no.	Size (nt)	GC content (%)	Similarity to OBE-1 (%)	GenBank accession no.	Size (nt)	GC content (%)	Similarity to OBE-1 (%)	GenBank accession no.
KM-2/Br/06	6,869	35	92.7	LC552049	4,309	37	89.4	LC552048	856	45	95.2	LC552047
FI-1/Br/08	6,867	35	93.0	LC552052	4,308	38	96.8	LC552051	867	45	98.4	LC552050

The sequences determined in this study will be useful in identifying the virulence factors implicated in bovine postnatal encephalomyelitis.

### Data availability.

The sequences are available in GenBank under the accession numbers LC552047.1 (KM-2/Br/06 S segment), LC552048.1 (KM-2/Br/06 M segment), LC552049.1 (KM-2/Br/06 L segment), LC552050.1 (FI-1/Br/08 S segment), LC552051.1 (FI-1/Br/08 M segment), and LC552052.1 (FI-1/Br/08 L segment).
